# Mental health during the Covid-19 pandemic: An international comparison of gender-related home and work-related responsibilities, and social support

**DOI:** 10.1007/s00737-024-01497-3

**Published:** 2024-09-05

**Authors:** Dominique Eugene, Jani Nöthling, Lorenzo Tarsitani, Christina Palantza, Davide Papola, Corrado Barbui, Richard Bryant, Catherine Panter-Brick, Brian J. Hall, Agnes Iok Fok Lam, Anja C. Huizink, Daniela Fuhr, Fredrick Dermawan Purba, Ellenor Mittendorfer-Rutz, Dhini Andriani, Judith van der Waerden, Ceren Acartürk, Gülşah Kurt, Sebastian Burchert, Christine Knaevelsrud, Anke B. Witteveen, Martina Patane, Soledad Quero, Amanda Díaz-García, Naser Morina, Irene Pinucci, Marit Sijbrandij, Soraya Seedat

**Affiliations:** 1 Faculty of Medicine and Health Sciences, Department of Psychiatry, Stellenbosch University, Cape Town, South Africa; 2 Department of Human Neurosciences, Sapienza University of Rome, Rome, Italy; 3 Vrije Universiteit Amsterdam, Amsterdam, the Netherlands; 4 Department of Global Health and Social Medicine, Harvard Medical School, Boston, MA USA; 5 WHO Collaborating Centre for Research and Training in Mental Health and Service Evaluation, Department of Neuroscience, Biomedicine and Movement Sciences, Section of Psychiatry, University of Verona, Verona, Italy; 6 Jackson School of Global Affairs, Yale University, New Haven, CT USA; 7 Department of Anthropology, Yale University, New Haven, CT USA; 8 Center for Global Health Equity, NYU Shanghai, Shanghai, China; 9 Department of Communication, Center for Macau Studies, University of Macau, Macau, China; 10 Department of Clinical, Neuro and Developmental Psychology and WHO Collaborating Center for Research and Dissemination of Psychological Interventions, Vrije Universiteit, Amsterdam, the Netherlands; 11 Department of Health Services Research and Policy, London School of Hygiene and Tropical Medicine, London, UK; 12 Department of Prevention and Evaluation, Leibniz Institute of Prevention Research and Epidemiology, Bremen, Germany; 13 University of Bremen, Health Sciences, Bremen, Germany; 14 Faculty of Psychology, Universitas Padjadjaran, Jatinangor, Indonesia; 15 Department of Clinical Neuroscience, Divions of Insurance medicine, Karolinska Institute, Solna, Sweden; 16 Institut Pierre Louis d’Epidémiologie et de Santé Publique, Social Epidemiology Research Team, Sorbonne Université, INSERM, Paris, France; 17 Department of Psychology, Koç University, Istanbul, Türkiye; 18 School of Psychology, UNSW, Sydney, New South Wales Australia; 19 Department of Education and Psychology, Division of Clinical Psychological Intervention, Freie Universität Berlin, Berlin, Germany; 20 Department of Basic, Clinical Psychology and Psychobiology, Universitat Jaume I, Castellón, Spain and CIBER de Fisiopatología de La Obesidad y Nutrición (CIBEROBN), Carlos III Institute of Health, Madrid, Spain; 21 Department of Psychology and Sociology, Universidad de Zaragoza (Campus Teruel), Teruel, Spain; 22 Department of Consultation-Liaison Psychiatry and Psychosomatic Medicine, University Hospital of Zurich, University of Zurich, Zurich, Switzerland; 23 Population Health Sciences, University of Bristol, Bristol, UK; 24 South African Medical Research Council/Stellenbosch University Genomics of Brain Disorders Research Unit, Department of Psychiatry, Cape Town, South Africa; 25 Gender and Health Research Unit, South African Medical Research Council, Cape Town, South Africa; 26 Institute for Social Innovation Fellow, Fielding Graduate University, Santa Barbara, CA USA; 27 HBNU Fogarty Global Health Training Program, Harvard T.H. Chan School of Public Health, Boston, MA USA

**Keywords:** Mental health, Anxiety, Depression, PTSD, home and work-related responsibilities, COVID-19

## Abstract

**Purpose:**

To assess gender differences in COVID-19 related changes in home and work responsibilities longitudinally, and determine whether these differences, together with other potential risk and protective factors, are associated with depression, anxiety, and post-traumatic stress disorder (PTSD) symptomatology.

**Method:**

Symptoms of depression, anxiety, and PTSD were measured using an online survey instrument, between May 2020 and April 2021, in four waves completed at 3-monthly intervals. Analyses were based on data from the COvid MEntal healTh (COMET) survey which investigated the mental health effects of the COVID-19 outbreak spanning 13 countries on five continents in N = 7,909 participants.

**Results:**

From the first to the last wave, women reported a greater increase in home and work responsibilities, and had higher depression, anxiety and PTSD scores compared to men. Women who reported a reduction in income due to the pandemic had higher depression scores. Working harder and experiencing a reduction in income were also associated with higher anxiety scores in women but not in men. Women were more likely to score above the cut-off for depression (32.5% vs 23.6%, *p* < .001), anxiety (21.2% vs 14.4%, *p* < .001) and PTSD (21.2% vs 14.4%, *p* < .001) than men during the first wave. Stronger reliance on socially supported coping mechanisms was a risk factor for depression, anxiety and PTSD in men and women.

**Conclusion:**

Women were more likely to report mental health problems which may be related to the gender disproportionate increase in home and work responsibilities but not necessarily due to COVID-19 stressors.

## Introduction

The COVID-19 pandemic had a major impact on the daily lives of individuals. It caused high levels of distress in individuals and their significant others who were infected with the virus and those who were financially or socially affected by it (Santomauro et al. 2021; Seedat and Rondon 2021). Millions of people worked from home and businesses closed, which had major economic impacts (Delardas et al. 2022; Richards et al. 2022). The full extent of the pandemic’s effects on mental health is still not fully known. The pandemic also exacerbated gender inequities in paid and unpaid work, with the burden of unpaid work (domestic, home schooling, and childcare) falling disproportionately on women, with substantial implications for their mental and physical health (Farré, 2022; Flor 2022). A survey among adults in Australia, during COVID-19-related restrictions, found that the higher risk of symptoms of anxiety and depression among women compared to men was, in part, explained by their disproportionate burden of unpaid caregiving (Hammarberg et al. 2020). Another study found that women reported greater concerns about their financial security which may have contributed to their mental health decline during the pandemic (Stallone 2021). However, this evidence is sparse and, as far as we are aware, there are no published cross-national studies.

As reported in several studies (Brooks et al. 2020; Huang et al. 2003; Maunder 2009; Peng et al. 2010) conducted during the most recent past viral epidemics (SARS and Ebola), women are more often in the role of having to create a comfortable atmosphere at home despite adversity. Daly and Robinson (2022) cautioned that although anxiety or depression was at its peak during the early stages of the pandemic, this may have been due to an acute reaction to an unexpected crisis rather than an increase in symptoms of depression and anxiety during COVID-19 (Daly and Robinson 2022). In Europe, particularly in France, the national public health agency reported that rates of depression and anxiety soared in February 2021 compared to previous years (Laham et al. 2021; France Santé publique, 2022). During the early phase of the pandemic, research in China related to the COVID-19 pandemic focused primarily on health care workers and reported increases in stress levels, anxiety, and depression (Lai et al. 2020). A study conducted in China on Weibo posts users (a social media platform) found that negative emotions (e.g., anxiety, depression and indignation) and sensitivity to social risks increased in more than 15,000 active users as a result of the outbreak (Li et al. 2020; Gan et al. 2022).

The COMET study examined the onset and development of mental health symptoms (anxiety, depression, PTSD) over time, in individuals from the general population across 13 countries in five continents, the Netherlands, Italy, Switzerland, Türkiye, Spain, Germany, France, United Kingdom, Sweden, South Africa, Indonesia, China, and Australia. At the time of data collection and in between lockdown implementation, several of these countries mandated strict sanitary measures such as wearing masks, social distancing, stay-at-home orders, remote working and curfews. These restrictions set the stage for widespread uncontrollable and unpredictable stressors related to the inability to access social support systems, fear of contamination, and financial loss (Cooke et al. 2020). The main objective of this study was to evaluate whether the course of mental health symptoms during the COVID-19 outbreak was predicted by demographic variables (age, gender, education level, profession, degree of economic losses), social isolation, level of exposure to the COVID-19 outbreak, pre-existing mental health problems, contamination fear, cultural value orientations, availability of social support, and coping strategies. In this paper we explored (1) gender differences in COVID-19 related changes in work and home responsibilities from the first to the last wave; (2) the association between change in work and home responsibilities during the pandemic and depression, anxiety and PTSD symptoms from the first to the last wave; and (3) the predictive weight of demographic variables, home and work-related variables, and psychosocial risk and protective factors on mental health outcomes in men compared to women, in the first wave.

## Methodology

### Study design

The COMET study followed a prospective follow-up design that included a large international cohort. Starting in May 2020, mental health symptoms were examined across four waves in the same participants. Wave 1 (May 2020) measured symptoms as soon as possible after the start of the epidemic, Wave 2 (September 2020) was completed 3 months after Wave 1, Wave 3 (December 2020) was completed 6 months after Wave 1, and Wave 4 (March 2021) was completed 9 months after wave 1. Potential predictors for the development of depression, anxiety and PTSD symptoms were examined and included demographic variables, increase in home responsibilities, increase in work responsibilities, loss of income, loss of jobs, contamination fear, substance use, social support, self-sufficient coping, socially supported coping, and avoidant coping. More details about this international study and the survey process are available online (https://osf.io/bgtsf/).

### Participants and procedure

A total of 8,013 participants above the age of 18 were recruited online through university mailing lists and different social networks such as Facebook, Instagram, Twitter, and were collected at four periods of time between May 2020 and April 2021. The online tool Survalyzer (www.survalyzer.com) was used to administer the survey which comprised a series of self-report questionnaires. Before commencing, participants were provided with information about the study and informed consent was obtained. Participation in the survey was voluntary and participants were able to withdraw at any time. No identifying information was collected during assessments, and all data were anonymized and encrypted. As completing the questionnaires may have resulted in mild transient distress, participants were provided with mental health resources. Most countries compensated participants with an entry draw for an opportunity to receive 50 euros, while in Germany vouchers were provided. Based on responses for the variable gender (which for this paper was limited to binary definitions of female and male), some participants were excluded, resulting in a final sample of 7,909 for this analysis and 20477 observations over time. The study was approved by the ethical review boards of participating countries, the Health Research Ethics Committee, the Swedish Ethical Review Authority, the Règlement Général sur la Protection des Données (RGPD) and the Informatique et Libertés law. The study was conducted according to the ethical guidelines and principles of the International Declaration of Helsinki, the South African Guidelines for Good Clinical Practice (2006), the Medical Research Council (MRC) Ethical Guidelines for Research (2002), and the Department of Health Ethics in Health Research: Principles, Processes and Studies (2015).

### Measures

The survey tool and self-report measures included in the analysis for this paper are described in Table 1.

**Table 1:** Description of measures and variables used in the study

Measure	Description	Timepoint/sadministered and Cronbach alpha	Variable/s	Source of Measure
Demographic questionnaire	The questionnaire asked about (1) age, (2) if the participant was in a relationship, (3) and if the participant completed some level of tertiary education. Relationship status was asked in reference to the past month. Responses to relationships status and completion of tertiary education were measured using a yes/no response option	Item 1 completed at wave 1. Item 2 and 3 completed at wave 1, 2 and 4	Age, relationship status, level of education	Not applicable
Home- and work-related responsibilities	The questionnaire asked about (1) employment status which was measured in wave 1 and participant indicated if they were employed at the start of the pandemic or unemployed (included students, homemakers, pensioners, those who were retired and those who were voluntary workers), (2) loss of a jobs, (3) a reduction in income,(4) working harder, and (5) an increase in home responsibilities. All time-dependent questions were asked in relation to the past month. All of the items were scored or recorded using a yes/no binary response option	Item 1 completed at wave 1. Item 2 – 4 completed at all waves. Item 5 completed at wave 1 and 2	Employment status, loss of jobs, loss of income, working harder, increase in home responsibilities	Not applicable
Patient Health Questionnaire (PHQ-9)	The PHQ-9 is a self-report measure that was used to screen for depressive symptoms. The PHQ-9 consists of 9 items. Item responses are measured on a 0–3 scale with total scores ranging from 0 to 27. Higher scores indicate more severe depression. For detection of clinically significant symptoms of depression, a cut-off score of 10 was used	Wave 1—4. Cronbach range 0.90—0.91	Depression symptoms and status	(Kroenke et al. 2001)
Generalised Anxiety Disorder 7 (GAD-7)	The GAD-7 is a seven-item measure of anxiety symptoms. The seven items are each scored on a 0–3 scale, with a total score range of 0 to 21. Higher scores indicate more severe anxiety symptoms. For detection of clinically significant levels of anxiety, a cut-off score of 10 was used	Wave 1- 4 Cronbach range 0.91—0.93	Anxiety symptoms and status	(Spitzer et al. 2006)
PTSD Checklist DSM-5 (PCL-5)	The 4-item PCL-5 scale was used to measure PTSD symptoms during the past week according to DSM-5 criteria for PTSD. The items assess symptoms on a 0–4 scale with total scores ranging between 0 and 16. Higher scores indicate more severe symptoms of PTSD. For detection of clinically significant levels of PTSD, a cut-off score of 10 was used	Wave 1 – 4. Cronbach range 0.82—0.83	PTSD symptom severity and status	(Blevins et al. 2015)
Substance Use Brief Screen (SUBS)	The SUBS is a four-item scale used to screen for past-year use of substances. Responses are measure on a 0–3 scale inquiring about how many days in the past year a participant used tobacco, alcohol, illicit drugs and prescription medication (recreational use). Higher scores indicate less frequent use of substances	Wave 1 and 4. Ordinal measure	Tobacco, alcohol, drug and recreational prescription medication use	(McNeely et al. 2015)
The Padua Inventory (PI)	The contamination obsessions and washing compulsions subscale of the PI was used to enquire about contamination fear. The 10 items were scored on a 1–5 scale with total scores ranging between 10 and 50. Higher scores indicate a more severe fear of contamination	Wave 1, 3 and 4. Cronbach range 0.91	Contamination fear	(Burns et al. 1996)
Brief-COPE	The Brief-COPE is a multidimensional measure of strategies used for coping or regulating cognitions in response to stressors. We selected 14 items from the following subscales: distraction (items 1 and 19), active coping (items 2 and 7), use of emotional support (items 5 and 15), use of instrumental support (items 10 and 23), venting (items 9 and 21), positive reframing (items 12 and 17) and planning (items 14 and 25). Responses were measured on a 0–3 scale inquiring about self-sufficient coping (range 0—18), socially supported coping (0—18) and avoidant coping (0—6). Higher scores indicate more reliance on a coping strategy	Wave 1 – 4. Cronbach range 0.87—0.91	Self-sufficient coping, socially supported coping and avoidant coping	(Carver et al. 1989)
Oslo Social Support Scale (OSSS-3)	The OSSS-3 measures levels of social support using three items. The items inquire about the number of people the respondent feels close to, the interest and concern shown by others, and the ease of obtaining practical help from others. Total scores range between 3–14 with higher scores indicating greater support	Wave 1 – 4. Ordinal measure	Social support	(Kocalevent et al. 2018)

### Statistical analyses

All statistical analyses were conducted using IBM SPSS Statistics version 27 and R statistics. Variables were divided into (1) demographic factors which included age, being in a relationship and completing tertiary education; (2) home and work-related variables which included being employed/unemployed, experiencing an increase in home responsibilities, working harder, having a reduction in income and losing a job; (3) mental health outcomes which included depression, anxiety and PTSD cut-offs and continuous scores; (4) potential risk and protective factors which included contamination fear, substance use, social support, self-sufficient coping, socially-supported coping and avoidant coping. Country was not controlled for because of the unequal group sizes across countries. Descriptive statistics (n and %) were calculated to the determine the distribution of participants across countries and to determine the gender stratified percentages of participants who were in relationships, completed tertiary education, experienced an increase in home responsibilities, were employed/unemployed or lost their jobs, experienced working harder, experienced a reduction in income, or scored above cut-off for depression, anxiety and PTSD. Observations across waves were combined. An average score across the waves for each participant was not used. The average score across waves was compared for women to the average score across waves for men. Chi-square tests were used to assess differences between men and women for the aforementioned binary variables. Differences between men and women (mean and standard deviation) for the continuous variables age, depression, anxiety, PTSD, contamination fear, substance use, social support, self-sufficient coping, socially supported coping and avoidant coping were determined using ANOVAs. Bonferroni correction was applied for multiple testing with an adjusted p value of 0.010.

Factorial ANOVAs, controlling for mental health scores in different waves, were used to determine if there were differences in depression, anxiety and PTSD scores between women and men based on home- and work-related variables. Cohen’s d was calculated to determine effect sizes.

Demographic, home, work, and other risk and protective factors were combined in the regression models. The combination of variables was not measured at all timepoints. As such, only wave one contained a complete set of data for all variables and the regression analysis was limited to this timepoint. There were no missing data in wave one. Linear regression modelling was used with six models computed, one for each outcome (depression, anxiety and PTSD) and stratified by gender (women and men). To compare the regression path coefficients between women and men for each of the three outcomes, the multi-group analysis (MGA) comparison feature of partial least square structural equation modeling (PLS-SEM) was used.

## Results

### Descriptive statistics

There were 7,516 participants who reported currently living in one of the thirteen countries included in the study (see Table 2).

**Table 2:** Distribution of participants across countries (residence at the time of the survey)

Country	Number of Participants	Percentage of Total Participants
Italy	1,381	17.6
China (Macau SAR)	729	9.3
Australia	717	9.1
Turkey	681	8.7
France	675	8.6
Germany	620	7.9
The Netherlands	596	7.6
Indonesia	593	7.5
South Africa	580	7.4
Spain	382	4.9
Sweden	288	3.7
Switzerland	143	1.8
United Kingdom	131	1.7
Total	7,516	

### Demographic and clinical comparison between men and women

The mean age of participants was 40.31 years (*SD* = 15.43, range = 18–95). The majority of participants were in a relationship (64.5%), had completed tertiary education (78.1%) and were employed (64.7%). More than a third of participants reported an increase in home responsibilities (39.2%), while 26.9% reported a reduction in income, 23.4% reported working harder, and 16.1% reported losing a job. Men who participated in the study were slightly older than women (41.57 vs 40.02, *p* < 0.001) and were more likely to be in a relationship (66.7% vs 62.2%, *p* < 0.001) compared to women. Women were more likely to report an increase in home responsibilities (40.8% vs 37.8%, *p* = 0.004) and working harder (24.4% vs 22.3%, *p* = 0.004) compared to men. There were no significant differences between men and women for tertiary education completed, loss of employment, and reduction in income.

When considering clinical characteristics, women were more likely to score above cut-off for depression (32.5% vs 23.6%, *p* < 0.001), anxiety (21.2% vs 14.4%, *p* < 0.001) and PTSD (21.2% vs 14.4%, *p* < 0.001) than men. Women also reported higher scores for depression (*M* = 8.12 vs *M* = 6.68, *p* < 0.001), anxiety (*M* = 6.03 vs *M* = 4.79, *p* < 0.001) and PTSD (*M* = 4.02 vs *M* = 3.33, *p* < 0.001) compared to men. Women scored higher on contamination fears (*M* = 22.43 vs *M* = 20.90, *p* < 0.001) and substance use, where lower scores indicate more substance abuse (*M* = 10.58 vs *M* = 10.22, *p* < 0.001). Women also endorsed more social support (*M* = 12.23 vs *M* = 11.76, *p* < 0.001), self-sufficient coping (*M* = 9.81 vs *M* = 9.31, *p* < 0.001), socially supported coping (*M* = 8.11 vs *M* = 6.62, *p* < 0.001) and avoidant coping (*M* = 3.22 vs *M* = 2.68, *p* < 0.001) compared to men (see Table 3 for details).

**Table 3:** Comparison of differences between men and women on demographic and clinical variables

	All	Women	Men	Gender comparison	
	n (%)	n (%)	n (%)	*X*^*2*^	*p*
In a relationship	16087 (100)	12557 (100)	3530 (100)	23.76	< 0.001**
Yes	10170 (63.2)	7815 (62.2)	2355 (66.7)		
No	5917 (36.8)	4742 (37.8)	1175 (33.3)		
Tertiary education completed	7396 (100)	5627 (100)	1769 (100)	0.334	0.564
Yes	5768 (78.0)	4384 (77.9)	1384 (78.2)		
No	1628 (22.0)	1243 (20.5)	385 (21.8)		
Increase in home responsibilities	12356 (100)	9587 (100)	2769 (100)	8.43	0.004*
Yes	4962 (40.2)	3916 (40.8)	1046 (37.8)		
No	7394 (59.8)	5671 (59.2)	1723 (62.2)		
Employed	7803 (100)	5975 (100)	1828 (100)	6.78	0.009*
Yes	4978 (63.8)	3765 (63.0)	1213 (66.4)		
No	2825 (36.2)	2210 (37.0)	615 (33.6)		
Unemployed	17803 (100)	5975 (100)	1828 (100)	3.65	0.056
Yes	1885 (24.2)	1474 (24.7)	411 (22.5)		
No	5918 (75.8)	4501 (75.3)	1417 (77.5)		
Lost job	20075 (100)	15696 (100)	4379 (100)	0.29	0.591
Yes	3253 (16.2)	2555 (16.3)	698 (15.9)		
No	16822 (83.8)	13141 (83.7)	3681 (84.1)		
Working harder	20146 (100)	15755 (100)	4391 (100)	8.26	0.004*
Yes	4826 (24.0)	3846 (24.4)	980 (22.3)		
No	15320 (76.0)	11909 (75.6)	3411 (77.7)		
Reduced income	20045 (100)	15685 (100)	4360 (100)	2.52	0.112
Yes	5324 (26.6)	4125 (26.3)	1199 (27.5)		
No	14721 (73.4)	11560 (73.7)	3161 (72.5)		
Depression	19978 (100)	15475 (100)	4246 (100)	122.44	< 0.001**
Above cut-off	6124 (30.7)	5022 (32.5)	1003 (23.6)		
Below cut-off	13854 (69.3)	10453 (67.5)	3243 (76.4)		
Anxiety	20477 (100)	15817 (100)	4401 (100)	101.21	< 0.001**
Above cut-off	4064 (19.8)	3354 (21.2)	633 (14.4)		
Below cut-off	16413 (80.2)	12463 (78.8)	3768 (85.6)		
PTSD	20477 (100)	15817 (100)	4401 (100)	101.21	< 0.001**
Above cut-off	4064 (19.8)	3354 (21.2)	633 (14.4)		
Below cut-off	16413 (80.2)	12463 (78.8)	3768 (85.6)		
	All	Women	Men	Gender comparison	
	M (SD)	M (SD)	M (SD)	*F*	*p*
Age	40.31 (15.43)	40.02 (15.18)	41.57 (16.15)	56.82	< 0.001**
Depression	7.83 (6.18)	8.12 (6.22)	6.68 (5.78)	180.85	< 0.001**
Anxiety	5.77 (5.14)	6.03 (5.19)	4.79 (4.78)	208.12	< 0.001**
PTSD	3.88 (3.57)	4.02 (3.60)	3.33 (3.38)	134.26	< 0.001**
Contamination fear	22.10 (9.09)	22.43 (9.20)	20.90 (8.58)	86.68	< 0.001**
Substance use	10.50 (1.76)	10.58 (1.71)	10.22 (1.89)	83.15	< 0.001**
Social Support	12.12 (2.19)	12.23 (2.17)	11.76 (2.19)	156.21	< 0.001**
Self-sufficient coping	9.70 (4.24)	9.81 (4.20)	9.31 (4.38)	52.74	< 0.001**
Socially supported coping	7.79 (4.52)	8.11 (4.47)	6.62 (4.47)	386.70	< 0.001**
Avoidant coping	3.11 (1.65)	3.22 (1.63)	2.68 (1.66)	391.05	< 0.001**

### Gender, home- and work-related variables and mental health

Depression, anxiety and PTSD scores stratified by gender along with home and work-related interactions are reported in Table 4. There were no significant differences between men and women who did and did not report an increase in home responsibilities, working harder or losing a job for depression, anxiety and PTSD. There were also no significant differences between men and women who did and did not report a reduction in income for the outcomes of anxiety and PTSD.

**Table 4:** Differences in depression, anxiety and PTSD scores between men and women based on home and work-related variables

	Depression				Anxiety				PTSD			
	Women	Men	Gender comparison		Women	Men	Gender comparison		Women	Men	Gender comparison	
	M(SD)	M(SD)	*F*	*p*	M(SD)	M(SD)	*F*	*p*	M(SD)	M(SD)	*F*	*p*
Gender x Increase in home responsibilities			1.44	0.230			1.76	0.190			1.59	0.210
Yes	9.45 (6.52)	7.76 (6.10)			7.05 (5.4)	5.65 (5.08)			4.77 (3.79)	3.97 (3.65)		
No	7.86 (6.15)	6.52 (5.78)			5.43 (5.06)	4.27 (4.59)			3.54 (3.38)	2.96 (3.18)		
Gender x Working harder			0.00	0.970			0.60	0.440			0.72	0.400
Yes	8.32 (6.01)	7.00 (5.65)			6.58 (5.22)	5.29 (4.66)			4.22 (3.62)	3.57 (3.36)		
No	8.05 (6.28)	6.59 (5.82)			5.84 (5.17)	4.65 (4.81)			3.95 (3.58)	3.26 (3.39)		
Gender x Reduced income			5.18	0.020*			3.36	0.070			0.66	0.420
Yes	9.59 (6.72)	8.41 (6.51)			7.25 (5.67)	5.92 (5.33)			4.90 (3.88)	4.18 (3.82)		
No	7.59 (5.94)	6.02 (5.34)			5.59 (4.94)	4.35 (4.49)			3.70 (3.43)	3.01 (3.15)		
Gender x Lost job			2.34	0.130			1.89	0.170			0.53	0.470
Yes	9.51 (6.95)	7.79 (6.64)			7.24 (5.67)	5.87 (5.52)			4.96 (4.01)	4.13 (3.91)		
No	7.84 (6.03)	6.47 (5.57)			5.79 (5.06)	4.59 (4.61)			3.83 (3.48)	3.18 (3.25)		

There was a significant difference between men and women who did and did not report a reduction in income for the outcome depression *F*(1, 18,747) = 5.18, *p* = 0.020. Women who reported a reduction in income had the highest scores for depression (*M* = 9.59, *SD* = 6.69). Women who reported a reduction in income also had significantly higher depression scores compared to women who did not report a reduction in income (*M* = 7.59, *SD* = 5.94, *p* < 0.001, *Cohen’s d* = 0.33). Men who reported a reduction in income had significantly higher depression scores (*M* = 8.41, SD = 6.51) compared to men who did not (*M* = 6.02, *SD* = 5.34, *p* < 0.001, *Cohen’s d* = 0.42). Women who reported a reduction in income had a significantly higher depression score compared to men who reported a reduction in income (*p* < 0.001, *Cohen’s d* = 0.18).

### Gender and predictors of mental health outcomes

The findings of regression models investigating demographic variables, home- and work-related variables, and potential psychosocial risk and protective variables as predictors of depression, anxiety and PTSD are presented in Table 5 and Table 6. Findings were stratified by gender. Table 7 presents the gender comparison of standardized beta values (regression path coefficients) resulting from the regression models.

**Table 5:** Predictors of depression, anxiety and PTSD stratified by gender

			Women					Men		
	Std *B*	Std Err	*p*	Lower	Lower	Std *B*	Std Err	*p*	Lower	Upper
Depression										
In a relationship	0.11	0.15	< 0.001**	1.05	13.64	0.12	0.26	< 0.001**	0.94	1.97
Tertiary education	0.11	0.18	< 0.001**	1.33	2.03	0.07	0.30	< 0.001**	0.45	1.62
Home responsibilities	0.11	0.15	< 0.001**	1.09	1.68	0.08	0.25	< 0.001**	0.51	1.51
Working harder	0.02	0.18	0.060	-0.01	0.68	0.02	0.30	0.311	-0.29	0.91
Lost job	0.10	0.24	< 0.001**	1.41	2.37	0.10	0.42	< 0.001**	0.10	1.02
Reduced income	0.05	0.16	< 0.001**	0.29	0.93	0.09	0.28	< 0.001**	0.55	1.63
Contamination fear	0.19	0.01	< 0.001**	0.11	0.14	0.20	0.01	< 0.001**	0.11	0.16
Substance use	-0.16	0.04	< 0.001**	-0.67	-0.50	-0.16	0.06	< 0.001**	-0.61	-0.35
Social support	-0.06	0.03	< 0.001**	-0.24	-0.10	0.00	0.06	0.924	-0.11	0.12
Self-sufficient coping	-0.26	0.02	< 0.001**	-0.44	-0.35	-0.27	0.04	< 0.001**	-0.44	-0.30
Socially supported coping	0.16	0.02	< 0.001**	0.18	0.25	0.22	0.04	< 0.001**	0.22	0.36
Avoidant coping	0.20	0.05	< 0.001**	0.68	0.88	0.17	0.08	< 0.001**	0.45	0.78
Anxiety										
In a relationship	0.04	0.13	< 0.001**	0.18	0.68	0.06	0.22	< 0.001**	1.10	-0.22
Tertiary education	0.10	0.15	< 0.001**	0.97	1.56	0.08	0.25	< 0.001**	0.43	1.42
Home responsibilities	0.1	0.13	< 0.001**	0.77	1.27	0.09	0.22	< 0.001**	0.44	1.29
Working harder	0.07	0.15	< 0.001**	0.55	1.14	0.04	0.26	0.069	-0.98	0.04
Lost job	0.08	0.21	< 0.001**	0.08	0.91	0.09	0.36	< 0.001**	0.71	2.11
Reduced income	0.04	0.14	0.003*	0.14	0.69	0.03	0.23	0.151	-0.12	0.80
Contamination fear	0.24	0.01	< 0.001**	0.12	0.14	0.24	0.01	< 0.001**	0.11	0.15
Substance use	-0.14	0.04	< 0.001**	-0.49	-0.34	-0.13	0.05	< 0.001**	-0.44	-0.22
Social support	-0.04	0.03	< 0.001**	-0.15	-0.04	0.02	0.05	0.351	-0.05	0.14
Self-sufficient coping	-0.23	0.02	< 0.001**	-0.33	-0.26	-0.26	0.03	< 0.001**	-0.35	-0.23
Socially supported coping	0.16	0.02	< 0.001**	0.16	0.22	0.24	0.03	< 0.001**	0.21	0.33
Avoidant coping	0.18	0.04	< 0.001**	0.48	0.65	0.13	0.07	< 0.001	0.25	0.53
PTSD										
In a relationship	0.08	0.09	< 0.001**	0.44	0.79	0.09	0.16	< 0.001**	0.36	0.98
Tertiary education	0.09	0.10	< 0.001**	0.57	0.98	0.07	0.18	0.001*	0.23	0.93
Home responsibilities	0.12	0.09	< 0.001**	0.66	1.00	0.09	0.15	< 0.001**	0.34	0.94
Working harder	0.02	0.10	0.095	-0.03	0.37	0.01	0.18	0.585	-0.26	0.45
Lost job	0.07	0.14	< 0.001**	0.49	1.04	0.07	0.25	0.002*	0.27	1.24
Reduced income	0.03	0.10	0.011*	0.06	0.43	0.05	0.16	0.027*	0.04	0.05
Contamination fear	0.23	0.01	< 0.001**	0.10	0.12	0.28	0.01	< 0.001**	0.09	0.13
Substance use	-0.14	0.02	< 0.001**	-0.34	-0.25	-0.14	0.04	< 0.001**	-0.33	-0.18
Social support	-0.03	0.02	0.014*	-0.09	-0.01	0.01	0.03	0.632	-0.05	0.08
Self-sufficient coping	-0.19	0.01	< 0.001**	-0.19	-0.14	-0.21	0.02	< 0.001**	-0.21	-0.12
Socially supported coping	0.13	0.01	< 0.001**	0.08	0.13	0.20	0.02	< 0.001**	0.20	0.20
Avoidant coping	0.18	0.03	< 0.001**	0.35	0.46	0.14	0.05	< 0.001**	0.20	0.39

**Table 6:** Summary statistics of multiple regression models stratified by gender

Model	*R*^*2*^	Δ* R*^*2*^	*F*	*df1*	*df2*	*p*
Women Depression	0.24	0.24	140.93	12	5451	< 0.001**
Men Depression	0.26	0.26	51.39	12	1717	< 0.001**
Women Anxiety	0.21	0.21	120.80	12	5449	< 0.001**
Men Anxiety	0.23	0.23	43.21	12	1717	< 0.001**
Women PTSD	0.23	0.23	136.95	12	5451	< 0.001**
Men PTSD	0.25	0.24	47.68	12	1716	< 0.001**

**Table 7:** Comparison of standardized beta scores between men and women

	Women Std *B*	Men Std *B*	*p*	Abs Diff *p*
Depression				
In a relationship	-0.11	-0.12	0.346	0.673
Tertiary education completed	-0.11	-0.07	0.926	0.156
Increase in home responsibilities	0.11	0.08	0.120	0.244
Working harder	0.02	0.02	0.510	0.959
Lost job	0.10	0.10	0.485	0.975
Reduced income	0.05	0.09	0.946	0.107
Contamination fear	0.19	0.20	0.703	0.628
Substance use	-0.16	-0.16	0.605	0.828
Social support	-0.06	0.00	0.992	0.015*
Self-sufficient coping	-0.26	-0.27	0.396	0.805
Socially supported coping	0.15	0.22	0.983	0.026*
Avoidant coping	0.20	0.17	0.143	0.280
Anxiety				
In a relationship	-0.04	-0.06	0.178	0.372
Tertiary education completed	-0.10	-0.08	0.821	0.372
Increase in home responsibilities	0.10	0.09	0.322	0.648
Working harder	0.07	0.04	0.106	0.238
Lost job	0.08	0.09	0.599	0.813
Reduced income	0.04	0.03	0.427	0.859
Contamination fear	0.24	0.23	0.489	0.988
Substance use	-0.14	-0.13	0.593	0.793
Social support	-0.04	0.02	0.993	0.037*
Self-sufficient coping	-0.23	-0.26	0.210	0.432
Socially supported coping	0.16	0.25	0.995	0.008*
Avoidant coping	0.18	0.13	0.059	0.115
PTSD				
In a relationship	-0.08	-0.09	0.383	0.771
Tertiary education completed	-0.09	-0.07	0.811	0.378
Increase in home responsibilities	0.12	0.09	0.141	0.336
Working harder	0.02	0.01	0.348	0.743
Lost job	0.07	0.07	0.491	0.957
Reduced income	0.03	0.05	0.735	0.501
Contamination fear	0.28	0.28	0.479	0.976
Substance use	-0.14	-0.14	0.493	0.980
Social support	-0.03	0.01	0.933	0.127
Self-sufficient coping	-0.19	-0.20	0.272	0.508
Socially supported coping	0.13	0.20	0.982	0.028*
Avoidant coping	0.18	0.14	0.066	0.126

#### Demographic predictors, gender and mental health outcomes

For women and men, not being in a relationship was associated with increased scores for depression (women *B* = 1.35, *p* < 0.001, men *B* = 1.46, *p* < 0.001), anxiety (women *B* = 0.43, *p* < 0.001, men *B* = 0.66, *p* < 0.001) and PTSD (women *B* = 0.62, *p* < 0.001, men *B* = 0.67, *p* < 0.001). Similarly, for women and men, not having completed tertiary education was associated with increased scores for depression (women *B* = 1.68, *p* < 0.001, men *B* = 1.04, *p* < 0.001), anxiety (women *B* = 1.26, *p* < 0.001, men *B* = 0.92, *p* < 0.001) and PTSD (women *B* = 0.78, *p* < 0.001, men *B* = 0.58, *p* < 0.001). The standardized regression coefficients for being in a relationship and completing tertiary education did not differ between women and men.

#### Home- and work-related predictors, gender and mental health outcomes

Reporting an increase in home responsibilities was associated with an increase in depression (women *B* = 1.39, *p* < 0.001, men *B* = 1.01, *p* < 0.001), anxiety (women *B* = 1.02, *p* < 0.001, men *B* = 0.87, *p* < 0.001) and PTSD (women *B* = 0.83, *p* < 0.001, men *B* = 0.64, *p* < 0.001) scores in both women and men. Working harder was not associated with increased depression or PTSD scores in women or men. Working harder was associated with increased anxiety scores in women (*B* = 0.84, *p* < 0.001), but not in men. Job loss was associated with an increase in depression (women *B* = 1.89, *p* < 0.001, men *B* = 1.84, *p* < 0.001), anxiety (women *B* = 1.32, *p* < 0.001, men *B* = 1.41, *p* < 0.001) and PTSD (women *B* = 0.76, *p* < 0.001, men *B* = 0.76, *p* < 0.001) scores in both women and men. Reduced income was associated with an increase in depression (women *B* = 0.61, *p* < 0.001, men *B* = 1.09, *p* < 0.001) and PTSD (women *B* = 0.24, *p* = 0.011, men *B* = 0.36, *p* = 0.027) scores in women and men. Reduced income was also associated with an increase in anxiety scores in women (*B* = 0.41, *p* = 0.003), but not in men. The standardized regression coefficients for an increase in home responsibilities, working harder, losing a job, and reporting a reduction in income did not differ significantly between women and men.

#### Psychosocial risk and protective factors, gender and mental health outcomes

Increased contamination fear scores were associated with increased depression (women *B* = 0.13, *p* < 0.001, men *B* = 0.14, *p* < 0.001), anxiety (women *B* = 0.13, *p* < 0.001, men *B* = 0.13, *p* < 0.001) and PTSD (women *B* = 0.11, *p* < 0.001, men *B* = 0.11, *p* < 0.001) scores in both women and men. Higher substance use was associated with increased depression (women *B* = -0.59, *p* < 0.001, men *B* = -0.48, *p* < 0.001), anxiety (women *B* = -0.41, *p* < 0.001, men *B* = -0.33, *p* < 0.001) and PTSD (women *B* = -0.29, *p* < 0.001, men *B* = -0.26, *p* < 0.001) scores in both women and men. The standardized regression coefficients for contamination fear and substance use did not differ significantly between women and men.

Decreased social support was associated with increased depression (*B* = -0.17, *p* < 0.001), anxiety (*B* = -0.10, *p* < 0.001) and PTSD (*B* = -0.05, *p* = 0.014) scores in women, but not in men. The standardized regression coefficients for social support differed significantly between women and men for depression (*p* = 0.015) and anxiety (*p* = 0.37) but not for PTSD. A larger effect was observed in women.

## Discussion

The key findings of this study can be summarized as follows. Higher rates of depression, anxiety and PTSD were observed in women compared to men across 13 countries during the COVID-19 pandemic. In addition, women who reported a reduction in income due to the pandemic had higher depression scores. Working harder and experiencing a reduction in income were also associated with higher anxiety scores in women, but not in men. Higher rates of common mental health problems are in line with prior studies indicating that women are in general more likely to be diagnosed with depression and anxiety compared to men (GBD 2019 Diseases and Injuries Collaborators 2020; Maeng and Milad 2015; Salk et al. 2017; Seedat et al. 2009). A review of global prevalence rates of depression and anxiety disorders recorded shortly before the onset of COVID-19 and nearly a year into the pandemic, found a 27.6% increase in depression and a 25.6% increase in anxiety disorders, with women being disproportionately affected (Santomauro et al. 2021; Dal Santo et al. 2022). The gender discrepancy in prevalence rates may be due to gender specific underlying biological drivers of depression and anxiety, higher exposure rates to interpersonal violence, and less reluctancy among women to disclose symptoms of mental health or to seek treatment (Li and Grahams 2016; McLean et al. 2011; Li et al. 2023). It may also be related to gender norms and inequality in household responsibilities and employment-related factors (Liu et al. 2021). It should be noted that despite the pandemic, women historically have been more likely to be diagnosed with depression and anxiety (Albert 2015; McLean et al. 2011; Salk et al. 2017; Santomauro et al. 2021).

Historically, women have shouldered a disproportionate number of responsibilities that include, but are not limited to, domestic chores (e.g., child rearing, husband rearing), educational endeavors, and professional aspirations, while attempting to find time to engage in self-care (Seedat and Rondon 2021). When considering home- and work-related differences between women and men, we found that women were more likely to report an increase in home responsibilities and to working harder compared to men. No differences in reduction in income or loss of employment was found between women and men. However, women who experienced a reduction in income had significantly higher depression scores compared to women who did not experience a reduction in income and compared to men who did experience a reduction in income. Working harder and experiencing a reduction in income was a predictor for anxiety in women, but not in men. Although sharing of household responsibilities between men and women has increased in recent years, women are still more likely to bear the brunt when it comes to tending to children and running the household (Carli 2020; Carlson et al. 2022). These responsibilities were exaggerated during the COVID-19 pandemic given school closures and being forced to work from home (Carli 2020). Women were also more likely to be employed as essential workers (e.g. nurses, social workers, health care administrative assistants and food service-related occupations) during the pandemic which, in addition to the increase in home obligations, may have also heightened work-related responsibilities. It is likely that the inequalities resulting from the pandemic placed women at greater risk for depression and anxiety (Chitiga et al. 2022; Seedat and Rondon 2021; Stallone 2021).

When considering demographic factors, not being in a relationship and not completing tertiary education were predictors of depression, anxiety and PTSD with similar effects observed between men and women. Loneliness, in general, but also during the pandemic, was associated with an increase in depression and anxiety symptoms and it is likely that loneliness resulting from social isolation and lockdowns had a larger effect on women and men who were not in a relationship or did not share a household with others (Beutel et al. 2017; Killgore et al. 2020; Laham et al. 2021; Okruszek et al. 2020; Li et al 2023). Individuals with a lower education level may have been at a higher risk of losing employment or a reduction in income during the pandemic. Individuals with a higher level of education may have also been better equipped to work remotely (M. C. Daly 2020; Kugler et al. 2023). It is, therefore, possible that women and men with a tertiary qualification were somewhat protected against the symptoms of depression and anxiety resulting from job losses and job insecurity (Ganson et al. 2021; Posel et al. 2021).

Consistent with prior findings, higher levels of contamination fears and substance use were a predictor of depression, anxiety and PTSD in women and men (Asmundson et al. 2020; Ganesan et al. 2021; Han et al. 2020). Lower social support was a predictor of increased depression and anxiety in women but not in men. Somewhat contrary to this, women and men who relied more on socially supported coping mechanisms reported higher depression, anxiety and PTSD scores with a larger effect observed for men. While social support is a known protective factor against adverse mental health outcomes in both women and men, they may differ in the way that they view social support (Gariépy et al. 2016; C. L. McLean et al. 2022; Li et al 2023). For example, men are more likely to perceive spousal support as social support while women perceive social support as a combination of spousal support and support from family and friends (Donato et al. 2018). Women are also more likely to report relying on socially supported coping mechanisms compared to men (C. L. McLean et al. 2022; Nolen-Hoeksema 2012; Theodoratou et al. 2023). Men who rely on socially supported coping mechanisms, and especially those who were not in a relationship, may have been particularly vulnerable to COVID-19 related stressors.

Similar effects were observed between women and men who relied on self-sufficient and avoidant coping. Relying on self-sufficient coping was associated with lower depression, anxiety and PTSD scores while relying on avoidant coping was associated with higher depression, anxiety and PTSD scores. This corresponds with prior findings where self-sufficient coping (e.g. planning, taking action and positive reframing) was generally considered a protective factor against adverse mental health outcomes and avoidant coping (self-distraction, denial, substance abuse, disengagement) was not (Costa et al. 2022; MacCann et al. 2022; Platte et al. 2022). In terms of novelty, this study adds to the field in addressing women’s perspective of social support, specifically the association of depression and anxiety with less social support in a multi-country, multi-cultural sample, and the link between increased home and work-related responsibilities and adverse mental health outcomes.

### Strengths and limitations

The study included a large sample of respondents from thirteen different countries, allowing for several potential risk and protective factors and adverse mental health outcomes to be modelled as well as allowing for stratification by gender. A few limitations warrant mention. There was a high dropout in successive waves. We did not have a pre-pandemic measure of depression, anxiety and PTSD and cannot compare pre- to post-pandemic changes in mental health, but rather symptom change over time. The regression analysis was limited to one time-point and causality cannot be inferred from the findings. Effect sizes were for the most part small and statistically significant differences may not signify clinically significant differences. Convenience sampling was used in the study which limits generalizability of the findings. Selection bias may have occurred given that participants needed access to the internet to complete the survey and online surveys may carry inherent risk of being biased since those interested in the topic of the research are more likely to take part in the survey. The sample may not be representative of the geographic, cultural, and economic diversity throughout the thirteen countries. Most participants had completed tertiary education (78.1%), so the survey is not representative of the whole population, where the percentage of tertiary graduates to be lower were expected. However, this could also be used as a strength, as people with lower levels of education are expected to earn less and be more exposed to societal stressors. On account of the initial time-sensitive nature of the study, lockdown restrictions, and risks associated with face-to-face physical contact interviews, an online survey was deemed to be the best mode of delivery.

## Conclusion

Higher rates of depression, anxiety and PTSD was observed in women compared to men. Our findings suggest that the increase in symptoms among women may have been a result of increased home- and work-related responsibilities. Contamination fears, substance use, and avoidant coping contributed a similar proportion of risk for adverse mental health outcomes in women and men, while self-sufficient coping had a similar protective effect. The findings provide insight into the underlying exacerbation of symptoms of depression, anxiety and PTSD experienced during the COVID-19 pandemic. Future studies should address spouses’ perceptions and beliefs regarding gender roles and their contribution to stressors and mental health concerns among women; the ongoing impact of the pandemic on family and work life, evaluation of the work/family border theory (Clark 2000) which underlines the ability to compartmentalize and balance work and family; and the contribution of co-worker support to women and men in maintaining work /life balance.
